# Bilastine: A New Nonsedating Oral H1 Antihistamine for Treatment of Allergic Rhinoconjunctivitis and Urticaria

**DOI:** 10.1155/2013/626837

**Published:** 2013-07-14

**Authors:** Ole D. Wolthers

**Affiliations:** Asthma and Allergy Clinic, Children's Clinic Randers, Dytmærsken 9, 8900 Randers, Denmark

## Abstract

Bilastine is a new, well-tolerated, nonsedating H1 receptor antihistamine. In the fasting state bilastine is quickly absorbed, but the absorption is slowed when it is taken with food or fruit juice. Therefore, it is recommended that bilastine is taken at least one hour before and no sooner than two hours after a meal. Clinical studies sponsored by the manufacturer have shown that bilastine 20 mg once daily is as efficacious as other nonsedating antihistamines in allergic rhinoconjunctivitis and chronic urticaria in individuals from 12 and 18 years of age, respectively. Bilastine is efficacious in all nasal symptoms including obstruction and in eye symptoms. The observations indicate that non-sedating antihistamines, as opposed to what has been thought previously, may be helpful in patients with allergic rhinitis in whom nasal obstruction is a major concern. Current international guidelines need to be revised in the light of the recent evidence. Research into aspects of pharmacokinetics and efficacy and adverse effect profiles of bilastine in children under 12 years of age is needed as are dose-response assessments and studies planned rigorously with the aim of assessing quality of life effects.

## 1. Introduction

Current guidelines for diagnosis and treatment of allergic rhinoconjunctivitis and urticaria recommend nonsedating antihistamines as first line treatment [1, 2]. Though they are helpful in many patients with mild disease, the available non-sedating antihistamines may not be sufficiently efficacious in moderate to severe conditions [1, 2]. Therefore, the launch of a recently developed non-sedating oral antihistamine, *bilastine, *attracts attention [3]. The aim of this paper is to review the current evidence of bilastine in the treatment of allergic rhinoconjunctivitis and urticaria. 

### 1.1. Histamine and Allergy

Several mediators are involved in the pathophysiology; however, histamine plays a vital role in the allergic immediate reaction [4]. Once an allergen is introduced to Ig E-sensitized mast cells, a degranulation is triggered which causes histamine to be released. The effects of histamines are mediated through several receptors including H1, H2, H3, and H4 receptors that belong to the superfamily of G-protein-coupled receptors [5]. The biological effects of histamine in the allergic reaction are mediated through H1 receptors that coexist in active and inactive forms of g-protein-coupled receptors which balance each other. Histamine works as an agonist that pushes the balance to the active side leading to effects such as muscular contraction, bronchospasms, upregulation of endothelial permeability, and stimulation of sensory nerves and cough receptors [6]. H1 antihistamines work as inverse agonists that drive the balance toward the inactive side and suppress the effects of histamine. Since these effects are not genuine antagonistic but rather represent a balance displacement between active and inactive forms of H1 receptors, now, the term H1 antihistamine rather than the former “antihistamine antagonist” is used [7]. 

### 1.2. Chemical Structure and Pharmacodynamics

**Figure 1:**
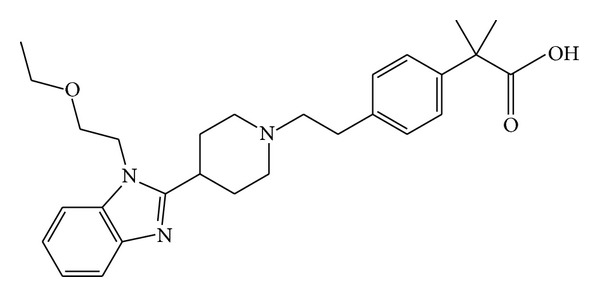
Chemical structure of bilastine: 2-[4-[2-[4-[1-(2-ethoxyethyl) benzimidazole-2-yl] piperidine-1-yl] ethyl] phenyl]-2-methylpropane acid.

Non-sedating antihistamines are part of a quite heterogeneous pharmacological group. Bilastine has not been derived structurally from other antihistamines. It belongs to the piperidine class of antihistamines as do loratadine, desloratadine, and fexofenadine (Figure 1). 

Like other antihistamines bilastine is an H1 receptor inverse agonist. *In vitro* studies have shown that bilastine has a high specific affinity for the H1-receptor but it has no or very low affinity for 30 other tested receptors [6]. The affinity for the H1 receptor is 3 and 6 times higher than for cetirizine and fexofenadine, respectively. *In vivo* studies in rats have demonstrated reduction in histamine-stimulated smooth muscular contraction, bronchospasms, endothelial permeability, and microvascular extravasation [8]. *In vivo* studies in the human population have demonstrated an inhibition of histamine-induced wheal and flare response activity of the skin which was marked with bilastine 20 mg as with cetirizine 10 mg [9]. 

### 1.3. Pharmacokinetics

In healthy adults, a mean oral systemic availability of bilastine of 61% has been reported [8]. In the fasting state bilastine is quickly absorbed, but the absorption is slowed when it is taken with food or fruit juice. The reduction seems to be due to a downregulation of the cell transport activity in the intestinal mucosa, the so-called organic anion-transporting polypeptides (OATP1A2) [10]. Therefore, it is recommended that bilastine is administered at least one hour before or no sooner than two hours after a meal [2, 10]. The maximum plasma concentration (220 ng/mL) of bilastine 20 mg was found 1.3 hours after administration, half time was 14.5 hours, and plasma protein binding was 84–90% [11]. Bilastine does not undergo any significant metabolization. Approximately, 95% is excreted intact in faeces (67%) or in urine (33%) [11]. Bilastine does not have any impact on the P450 (CYP) enzyme system of the liver, and there is no evidence of interaction with other drugs except that there is an increased uptake of bilastine when it is taken concomitantly with ketoconazole, erythromycin, or diltiazem [11]. This has been explained by probable interactions with intestinal transporters. Important pharmacokinetic parameters for non-sedating antihistamines and recommended doses in patients ≥12 years of age are listed in Table 1 [10–13]. There is little evidence, if any, that pharmacokinetic differences between specific drugs are important in clinical use.

## 2. Methodology

A PubMed literature search from January 1, 2000, through April 1, 2013, was conducted to track down randomized controlled studies of clinical efficacy of bilastine. This was supplemented with additional papers on bilastine and abstracts cited in reference lists, obtained from online sources, or supplied by Berlin-Chemie A. Menarini Aps, Hilleroed, Denmark. 

## 3. Results

The literature search revealed 2 efficacy studies in allergic rhinoconjunctivitis [14, 15], 1 in perennial rhinitis [16], and 1 in chronic idiopathic urticaria [17]. An overview of the studies is presented in Table 2. All studies were sponsored by the inventor and manufacturer of the drug FAES FARMA, S.A., Bilbao, Spain [14–17].

### 3.1. Allergic Rhinoconjunctivitis

As seen in Table 2, 3 large studies (*n* = 650–721) of oral bilastine 20 mg have been performed on symptomatic 12–70-year-old subjects. The studies have uniformly randomized, double-blinded, double-dummy, parallel-group, placebo-controlled, comparator-controlled, multicenter, and multinational study designs [14–16]. Two of the studies were conducted in Europe [14, 15], and one was conducted in Europe, South America, and South Africa [16]. Two of the studies were on subjects with seasonal pollen allergy [14, 15], and one study was on subjects with dust mite allergy and perennial rhinoconjunctivitis [16]. Bilastine was compared with the active drugs desloratadine 5 mg [14] and cetirizine 10 mg [15, 16]. In one study the blinded medicine was taken in a fasting state 1-2 hours before breakfast [14]; in the other studies it was taken one hour before or two hours after the breakfast [15, 16]. In the two studies with seasonal allergic rhinoconjunctivitis of two weeks duration treatment, compliance was approximately 100% in all treatment groups [14, 15]. Compliance was approximately 96% in all treatment groups in the perennial rhinitis study [16]. 

 The primary efficacy parameter in the studies was the area under the curve for total nasal symptom score (nasal obstruction, rhinorrhoea, sneezing, and nasal itching) and total nonnasal symptom score which in all studies included scores of itchy eyes, burning eyes, and reddening of eyes [14–16]; however, one of the studies also included the symptoms of foreign body sensation in the eyes, lacrimation, and itchy ears and/or palate [15]. Each of these symptoms was assessed over the preceding 12 hours twice daily, graded on a severity scale of 0–3. Compared to baseline values, the area under curve for total symptom score after two weeks treatment with placebo, bilastine, or desloratadine was reduced by 37.4%, 48.9%, and 49.5% (ANOVA; *P* = 0.02) in one study [14] and 47.4 (placebo), 65.2 (bilastine), and 71.5% (cetirizine) (ANOVA; *P* < 0.001) in another study [15]. In both studies the symptom-relieving effect of bilastine 20 mg was found to be statistically significant and comparable with the effect of desloratadine 5 mg and cetirizine 10 mg, respectively. Various *secondary efficacy parameters* supported these findings, and some of these have been republished separately for the evaluation of nasal obstruction [18] and eye symptoms [19]. Probably duplicate publications were made to further highlight the efficacy of bilastine on these specific symptoms since the evidence of efficacy of antihistamines on nasal obstruction and eye symptoms has been quite weak up to now.

 Surprisingly, in the third multinational study no statistical significant difference was observed in efficacy parameters during 4 weeks treatment with placebo, bilastine 20 mg, or cetirizine 10 mg [16]. A post hoc analysis, however, demonstrated statistically significant differences in symptom scores between the placebo groups, as subjects in South Africa scored significantly higher than subjects in Europe or South America. In addition, South Africans had higher basic scores than Europeans and South Americans; they had longer medical histories for perennial rhinitis; they weighed more and had a higher body mass index; the number of Caucasians among South Africans was statistically significantly lower than of Europeans and South Americans. Post hoc efficacy analyses of European and South American populations showed a statistically significant symptom reduction in the groups that received active treatment. Most important of all, perhaps, the study results threw light on the risk of bias in multinational studies. 

 A further study was conducted in 74 asymptomatic 18–55-year-old subjects with allergic rhinitis in a randomized, double-blinded, double-dummy, placebo-controlled, crossover study at a single European site [13] with the aim of estimating the time of onset and the duration of action of a single dose of bilastine 20 mg [13]. The study was conducted at a laboratory in which subjects were challenged with airborne grass allergens followed by active treatment. The primary efficacy parameter was nasal symptom score, which was assessed every 15 minutes for 6 hours and again 22–26 hours after administration. The symptom-relieving effect of bilastine and cetirizine was observed within one hour and could still be detected 26 hours later. The efficacy of fexofenadine 120 mg was comparable with bilastine and cetirizine for the first 6 hours, but it was statistically significantly lower after 22–26 hours. 

### 3.2. Chronic Urticaria

 Just one clinical study is available in urticaria (Table 2). That was a double-blinded, placebo-controlled, randomized, parallel-group, multinational study in 525 18–70-year-old subjects with chronic idiopathic urticaria [17]. Inclusion criteria were a documented history of chronic urticaria occurring ≥3 times/week for 6 weeks and an individual urticaria symptom score of ≥2 for two urticaria symptoms for ≥3 days during the 7 days screening period and at randomization. The urticaria symptom score was based on the severity of pruritus, the number of wheals, and the maximum size of wheals which were assessed daily in the morning and in the evening over the preceding 12 hour period (reflective) using 4 point scales of 0–3. The total symptom score was calculated as the sum of scores for pruritus, number of wheals, and wheal size. 

 The primary endpoint was mean change from baseline in the area under the curve in the patient reflective total urticaria symptom score. Similar comparable statistically significant symptom-suppressive effects of bilastine 20 mg (−115.21; confidence interval −123.95 to −106.47) and levocetirizine 5 mg (−125.50; confidence interval 134.63 to 118.35) daily for the 28 days assessments were found, both treatments being statistically significantly more effective than placebo (−81.50; confidence interval −90.19 to 72.81) (ANOVA; *P* < 0.001). Secondary efficacy outcome measures were investigator's symptom score and investigator's global clinical impression. Bilastine and levocetirizine were equally more effective than placebo in the secondary measures, and differences between the two active treatments were not seen. 

### 3.3. Quality of Life

Two studies of bilastine included secondary quality of life parameters [14, 17]. In the seasonal allergic rhinoconjunctivitis study the validated Rhinoconjunctivitis Quality of Life Questionnaire (RQLQ) was used and completed by the subjects [14, 20]. Seven domains were assessed in the questionnaire (activity limitation, emotional function, eye symptoms, nasal symptoms, nonnasal symptoms, practical problems, and sleep problems) on a scale of 0–6. Total RQLQ score was reduced by 1.3 in the placebo group and 1.6 in both the bilastine and cetirizine groups. Although the difference was statistically significant, it may be questioned as to which extent the difference may be relevant in real-life settings. That may apply also to secondary analyses in the chronic idiopathic urticaria study which found bilastine and levocetirizine improvements in the validated quality of life measures of Dermatology Life Quality Index (DLQI) and self-rated questionnaires for assessment of feeling of discomfort and quality of sleep [17]. 

### 3.4. Adverse Effects

Clinical studies in subjects with allergic seasonal rhinoconjunctivitis [14, 15], perennial rhinoconjunctivitis [16], and urticaria [17, 21] have consistently shown good tolerability with an incidence of treatment-related side effects (15–30%) at placebo levels (19–28%). The most common adverse effects were headache, somnolence, dizziness, and fatigue. Serious adverse effects were not reported. Generally, no variations in side effect profiles were observed between bilastine and comparable active treatments. One parallel-group study observed a statistically significant lower incidence of somnolence and fatigue in the bilastine group compared to the cetirizine group, but the number of patients who experienced these side effects was low (*n* = 1–17) [15]. It is not possible to draw any conclusions from the data and the findings have not been reproduced. 

Clinical experiences with the non-sedating antihistamine terfenadine identified a significant risk for death due to induced disturbances in the electrical conduction of the heart (prolonged QT interval). Terfenadine was withdrawn back in the 1990s. Since then, a lot of attention has been paid to the risk when new antihistamines are developed. A double-blinded, crossover study in 30 healthy subjects could not demonstrate any cardiac effects of bilastine 20 or 100 mg once daily [22].

### 3.5. Alcohol and Driving

It is well known that sedating antihistamines potentiate the impact of alcohol on somnolence, driving, and psychomotor functions [23]. Non-sedating antihistamines do not generally have the effect, although this has been reported by few patients [23, 24]. A double-blinded, placebo-controlled study assessed the influence of bilastine 20, 40, and 80 mg and the sedating anti-histamine hydroxyzine hydrochloride 25 mg on psychomotor functions, when the drugs were taken by healthy subjects with standardized alcohol consumption [25]. Psychomotor functions were measured with objective tests and subjective assessment. Statistically significant objective and subjective suppressions were observed for bilastine 80 mg; only subjective suppression was observed for bilastine 40 mg; a dose of 20 mg did not have any impact as compared to placebo. That was supported by another double-blinded, placebo-controlled study evaluating the test-driving effects of bilastine 20 and 40 mg after a single dose and after once-daily dosing for a week [26]. The primary efficacy endpoint was “standard deviation of lateral position” (SDLP), an objective measure of the ability to drive straight [27]. No effects were observed for the tested bilastine doses, neither after a single dose nor after once-daily dosing for a week. 

## 4. Discussion 

Clinical studies sponsored by the manufacturer of the drug have shown that bilastine 20 mg once daily is as effective as other non-sedating antihistamines for the treatment of seasonal allergic rhinoconjunctivitis and chronic idiopathic urticaria in children and adults from 12 and 18 years of age, respectively. Considering recent observations indicating that clinical trials sponsored by manufacturers more often than non-pharmaceutical company sponsored trials have favorable efficacy results it may be argued that further evaluations may be needed [28]. Certainly, the argument may be strengthened when part of the evidence is based on duplicate publications and post hoc analyses [16, 18, 19]. Further evidence testing is needed in patients with perennial rhinoconjunctivitis in whom the only available study so far failed to prove any effect on the primary efficacy outcome measure. Such evaluations should be conducted during short-term (weeks) as well as during intermediate-term (6–12 months) treatment. Having said that, there may be no reason to suspect that bilastine would not be as effective as other non-sedating antihistamines in perennial rhinoconjunctivitis [1, 2]. That is probably why bilastine has received registration also for perennial rhinoconjunctivitis despite the fact that in that specific group of patients the evidence has been based on post hoc secondary efficacy outcome measures [29]. Finally, the observation that bilastine was efficacious in nasal obstruction supports other recent findings that oral and intranasal non-sedating anti-H1 antihistamines, as opposed to what was previously thought, indeed, are helpful in patients in whom nasal obstruction is a major concern [30]. International guidelines may need to be revised in the light of this evidence [1]. 

 In the clinical management of allergic rhinoconjunctivitis and urticarial, conventional doses of non-sedating antihistamines in some patients have little effect and one may often end up with opting a dose increasing strategy which. Though such a strategy may be endorsed by current guidelines it is fair to say that as of yet it has little evidence [31]. Considering that a preliminarily published study on adult patients with urticaria found dose-related symptom-suppressive effects of bilastine 20, 40, and 80 mg, rigorous dose-response studies in patients with allergic rhinoconjunctivitis might be helpful [21]. 

 In the fasting state bilastine is quickly absorbed, but the absorption is slowed when it is taken with food or fruit juice. Therefore, it is recommended that bilastine is taken at least one hour before and no sooner than two hours after a meal. No other available non-sedating antihistamines have the restriction, and it is not known if the recommendation has any implication on the effect of bilastine on patients in real-life settings. Studies should be conducted to clarify that. Furthermore, there is a need to assess pharmacokinetics, efficacy, and side effects in separate populations of children including children younger than four years of age in whom modulations other than tablets often are more convenient. 

 In recent years increasing focus has been on comorbidity and general symptoms such as fatigue and quality of life deterioration in patients with seasonal and perennial allergic rhinoconjunctivitis [1, 2]. There is a need for well-planned clinical studies statistically powered to test bilastine effects on quality of life. That, however, applies to other non-sedating antihistamines as well.

## 5. Conclusions

Bilastine 20 mg once daily is as efficacious as other non-sedating antihistamines in allergic rhinoconjunctivitis and chronic urticaria. Bilastine is efficacious in all nasal symptoms including obstruction and in eye symptoms in patients with allergic rhinoconjunctivitis. Bilastine is well tolerated. In the fasting state bilastine is quickly absorbed, but the absorption is slowed when it is taken with food or fruit juice. Therefore, it is recommended that bilastine is taken at least one hour before and no sooner than two hours after a meal. International guidelines need to be revised in the light of the evidence of antihistamine effects on nasal obstruction. Research into pharmacokinetics, efficacy, and adverse effect profiles of bilastine in children under 12 years of age is needed as are dose-response assessments and studies planned rigorously with the aim of assessing quality of life effects.

## Figures and Tables

**Table 1:** Recommended doses in patients ≥12 years of age, pharmacokinetic properties, and discontinuation intervals prior to skin prick testing* for nonsedating antihistamines.

Generic name	Acrivastine	Cetirizine	Desloratadine	Ebastine	Fexofenadine	Levocetirizine	Loratadine	Bilastine
Dosage (mg × daily)	8 × 3	10 × 1	5 × 1	10–20 × 1	180 × 1	5 × 1	10 × 1	20 × 1
Rapid onset (h)	0.5–1	0.5–1	ND	1	1	0.5–1	0.5–1	0.5–1
Maximum effect (h)	1.5–2	4–6	ND	4–6	6	4–6	4–6	1.3-1
Duration of effect (h)	8–12	24	24	>24	24	24	24	>24
Metabolism (%)	20	<10	0	>90	0	<10	>90	0
Interactions	No	No	No	Yes	Yes	No	No	No
Discontinuation interval (d)	2	3	3	3	3	3	3	ND

ND: no data, based on the references [8, 14–16, 18, 19] and *on data in product summaries of the specific drugs.

**Table 2:** Randomized, double-blinded, parallel-group, placebo-controlled comparative studies of bilastine with other antihistamines: study characteristics and results of primary efficacy outcome measures.

Study	Indication	Primary outcome	Arm 1; *N*	Arm 2; *N*	Arm 3; *N*	Results for primary outcome
[14]	Seasonal allergic rhinitis	AUC for TSS from baseline ⇒ 14 days	Bilastine 20 mg; 233	Desloratadine 5 mg; 242	Placebo; 245	Bilastine significantly better than placebo Bilastine versus desloratadine: NS

[15]	Seasonal allergic rhinitis	AUC for TSS from baseline ⇒ 14 days	Bilastine 20 mg; 227	Cetirizine 10 mg; 228	Placebo; 226	Bilastine significantly better than placebo Bilastine versus cetirizine: NS

[16]	Perennial allergic rhinitis	AUC for TSS from baseline ⇒ 28 days	Bilastine 20 mg; 214	Cetirizine 10 mg; 217	Placebo; 219	Bilastine versus cetirizine and placebo: NS

[17]	Chronic idiopathic urticaria	Change in TSS from baseline ⇒ 28 days	Bilastine 20 mg; 173	Levocetirizine 5 mg; 165	Placebo; 184	Bilastine significantly better than placebo Bilastine versus levocetirizine: NS

AUC: area under curve; TSS: total symptom score; NS: not statistically significantly better.
